# Evaluation of an inter-professional training program for student clinical supervision in Australia

**DOI:** 10.1186/1478-4491-12-60

**Published:** 2014-10-14

**Authors:** Sue Gillieatt, Robyn Martin, Trudi Marchant, Angela Fielding, Kate Duncanson

**Affiliations:** School of Occupational Therapy and Social Work Faculty of Health Sciences, Curtin University, GPO Box U1987, Perth, Western Australia 6845 Australia

**Keywords:** Clinical placement, Clinical supervision, Clinical supervision training, Clinical supervisor, Student supervision

## Abstract

**Background:**

As a response to an Australian shortage of clinical health, nursing, and medical placements, Commonwealth Government funding has been directed to expand student training opportunities and increase the competence and number of available clinical supervisors. This paper evaluates the application of a particular supervision training model for this purpose. It considers the model’s suitability and relevance across professions and its impact on supervisory knowledge, skills, and values as well as the intention to supervise students.

**Methods:**

The design, delivery, and evaluation of a series of one-day introductory student clinical supervision training workshops for allied health disciplines, nursing, and medicine are considered. Participants evaluated Proctor’s model of clinical supervision, which was expanded by the trainers to incorporate diversity and power relations in student supervision.

**Results:**

Evaluation results suggest that adapting Proctor’s model for student clinical supervision is relevant across a broad range of health disciplines and clinical areas. Participants from 11 health professions reported that the training improved their knowledge, skills, and values and expanded their willingness to accept student clinical placements. The outcomes are suggestive of enhanced clinical supervision intent, capacity, and capability.

**Conclusions:**

The student supervision training improved participants’ confidence in their clinical supervision skills. The findings suggest that the training has the potential to extend capacity and capability for student supervision across health professions and in Health Workforce Australia’s identified priority areas of mental health, community health, rehabilitation, private practice, and non-government organisations. Findings also indicate that these gains are reliant on health organizations developing and sustaining cultures of learning.

## Background

The Australian Government created Health Workforce Australia (HWA) in 2008 to address chronic health workforce shortages [1]. HWA developed its Clinical Supervision Support Program (CSSP) to expand student placement opportunities, increase numbers of trained and competent clinical supervisors across health professions and settings, and encourage organizations to promote a positive culture of learning [2]. The primary foci of HWA’s CSSP included ensuring ‘clarity’ in the role and function of student supervision, creating ‘quality’ improvements, and developing a ‘culture’ which emphasises collaboration across professions and promotes an organizational learning culture. The CSSP sought to address the barriers of occupational stress and ‘workplace incivility and aggression’ [3] by resourcing and supporting clinical supervision training across the health workforce.

The implementation of HWA’s CSSP involved funding a variety of projects across Australia. One such project was undertaken by the Western Australian Department of Health (WADoH), which contracted three institutions, including Curtin University (Curtin), to provide student supervision training to allied health, nursing, and medical staff in government and non-government agencies, in both traditional areas and areas where numbers of student placements could be increased. Curtin’s training comprised five one-day introductory and three four-day advanced workshops. The one-day training provided an introduction to an expanded version of a supervision model often referred to as Proctor’s model [4], which has its origins in Kadushin’s work on social work supervision [5]. This paper evaluates the suitability of the supervision model and considers the implications of this training in increasing student placements and the number of competent student supervisors.

### Clinical supervision

Within health and human services literature, a variety of definitions, models, and applications of clinical supervision for staff and students are found [3, 6]. Despite the differences, common themes and strategies can be traced, such as one staff member (usually senior) providing supervision to a student, another staff member or intern for the purposes of quality client care, improved outcomes, efficiency, accountability, professional development, and support for the supervisee [7]. Inter-professional training within the clinical placement is also endorsed [2, 8–10].

Ideally, clinical supervision has three key functions: normative (administrative), formative (educative), and restorative (supportive) [3]. Kadushin, drawing on the work of Dalton from 1926, presented this approach for social workers in the US in 1976; Kadushin’s work was then adapted for counsellors in the UK by Proctor in 1987 [11], and Inskipp and Proctor in the 1990s [12]. Consequently, ‘Proctor’s model’ became widespread across health services [12] and clinical supervision was promoted by health departments in the UK [13, 14]. The model is frequently found within social work [4] and nursing [15], and recommended for use in medicine [16], while other allied health professions, such as physiotherapy, psychology, and speech therapy, report using discipline-specific supervisory models [17–19]. Given this diversity, this unique project sought to evaluate the suitability of an extended version of Proctor’s model for student clinical supervision training delivered in an inter-professional context.

A variety of terms are used to represent the three supervision functions of the model and this is represented in Table 1. While the three functions provide a focus for examining fundamental aspects of staff and student supervision, a key feature of the model is acknowledging and managing the conflicts that can exist between the functions. This means the difficulties and tensions associated with balancing the need to employ the accountability/normative, educative/formative, and supportive/restorative functions simultaneously are explored and addressed.

**Table 1:** **Kadushin and Proctor’s models and the three functions of clinical supervision**

Kadushin (1960s – 1990s)	Proctor (1980s – 2000s)	Function	Common-use language in this project (2013)
Managerial, Administrative	Normative	Managerial, administrative and evaluative to ensure standards, policies, and procedures are implemented and adhered to. Performance assessment and management when problematic.	Accountability
			Assessment
Educative	Formative	Professional development, teaching, and mentoring.	Learning style
Supportive	Restorative	Discussion to make sense of the emotional content of clinical practice so as to manage work-related stress.	Well-being
			Self-care

In some professions, such as nursing [20], psychology [21], physiotherapy [17], and more recently social work [22], these tensions have at times led to the three functions being separated in staff supervision. Such division has occurred where managerialist agendas dominate and the focus of supervision is on compliance with organizational and legislative requirements, usually within a line management relationship [20, 22]. In these situations, the underlying power relations are configured to privilege normative/accountability functions at the expense of the restorative/supportive and formative/educative functions which can result in supervisee dissatisfaction and potentially compromised supervision outcomes. In response, some professions have developed models of external supervision where responsibility for formative/educative and restorative/supportive functions are undertaken by an external supervisor while normative/accountability requirements are met through the organization [20–22]. While these models may be legitimate for staff supervision, they are inappropriate for the clinical supervision of students [2] where supervisors are accountable for assessment of student readiness for professional practice and it is therefore imperative that all three supervision functions are addressed simultaneously.

The selection of Proctor’s model for this project was also informed by factors other than its ability to address all three functions simultaneously; such as the applicability [3], transferability [23], and frequency of use of the model across health disciplines [3, 15]. However, while Proctor’s model addresses the three core supervision functions, contemporary clinical supervision practice suggests a need to attend to other factors [3, 4, 24]. This means expanding the model so that supervisors match the supervision tasks to the developmental stage of supervisees [6, 25] and work openly with power differentials in supervision relationships [4]. Quality supervision requires supervisors to be mindful of, work respectfully with, and critically reflect on human diversity such as culture, ethnicity, disability, socioeconomic status, religion, gender, sexuality, and life experiences [3, 4, 19, 26–28]. This issue is highly relevant in light of the increasing diversity of health consumers, students, and the health workforce [3].

These factors also highlight the complexity of providing quality clinical supervision to both students and staff and the need for a clinical supervisor to demonstrate and have access to a depth of knowledge, skills, and values. Supervisors require knowledge of supervisory models, standards and policies, supervisee developmental levels, adult learning principles, self-care frameworks, and the influence of diversity. Key skills and attributes include critical reflection, relationship-building, flexibility, assessment, managing problematic performance, providing communication and feedback, emotional literacy, applying professional boundaries, and demonstrating the capacity to create culturally safe environments. Values which promote quality supervision include honesty, integrity, respect, empathy, trust, openness, collaboration, appreciation of diversity, and high levels of self-awareness [3, 4, 6, 29, 30]. Since clinical supervision training facilitates the development of these attributes and has been found to promote improved client outcomes, supervisor competence, and supervisee job satisfaction [16, 21, 31–40], what does the literature say about the longer term implications for both student supervision training and broader workforce concerns?

### The relationship between clinical supervision and workforce concerns

The clinical supervision literature highlights that quality supervision impacts positively on the student placement experience which can have implications for the health workforce. Studies undertaken with student and new graduate nurses identified that recruitment and retention were strengthened when quality clinical supervision occurred [41–43]. For more experienced staff, clinical supervision is reported to increase job satisfaction, reduce stress, and enhance retention rates [35, 39, 44–46]. Cummins [13] reviewing 22 international studies in nursing concurred that regular clinical supervision improved job satisfaction and reduced stress but also found that it reduced burnout, enhanced therapeutic skills, promoted professional development, increased the sense of being supported, reduced professional isolation, and improved client care. These outcomes resulted in increased willingness to remain with the employing organization, particularly in the case of students and new graduates.

The priority for improving workforce distribution in Australia centres on the underserviced areas of rural and remote health, aged care, mental health, primary health care, community based services, and oral health, as well as the emerging private sector [2, 24]. The literature on the rural and regional health workforce suggests that these shortages can be partially addressed through clinical supervision for students and staff [7, 47–50]. Clinical supervision has also been shown to improve recruitment and retention in mental health and aged and community care [31, 42, 45, 51–55]. In private practice, clinical supervision has been found to promote staff wellbeing, job satisfaction, and competence – qualities identified as enhancing recruitment and retention [52].

## Methods

The one-day clinical supervision training program was developed for allied health, nursing, and medical staff with minimal experience in clinical supervision of students. An outline of the training program is provided in Table 2. The program was designed by three social workers with strong interest and experience in the development and delivery of training in clinical supervision. The three designers delivered the training and are experienced student and staff supervisors.

**Table 2:** **Outline of the one-day training program**

One-day clinical supervision training overview	
Morning session	• Defining the purpose, scope, and reach of clinical supervision
	• Supervision relationships, including:
	○ Identifying the key features of productive supervision relationships
	○ Consideration of the limits to supervision
	○ Exploration of boundaries in supervision
	○ Participants practising building supervision relationships
	○ Feedback from peers on the simulated activity
	• Learning styles and their influence in and on supervision, including participants exploring their learning style and the adjustments required in supervision when working with students with different learning styles
	• Domains of supervision introduced:
	○ Normative or administrative
	○ Formative or educative
	○ Restorative or supportive
	• Considering the use of power and authority in supervision and through the use of the supervision domains
Afternoon session	• Domains of supervision explored through:
	○ Discussion on participants’ experiences of supervision domains
	○ Consideration of how the domains intersect
	○ Participants practising implementing the domains of supervision
	○ Feedback from peers on the simulated activity
	• Respectfully acknowledging and working with diversity and difference in clinical supervision
	• Practising the provision of constructive feedback followed by feedback from peers on the simulated activity
	• Practising ‘authentic’ and ‘challenging’ supervision conversations, including informing a student their performance is not meeting the required standard; this was followed by feedback from peers on the simulated activity
	• Consolidation of learning with participants sharing ideas for their future supervision practice

The program utilized Proctor’s model and expanded it to attend to issues of diversity and power. Learning activities were positioned to reflect ‘real life’ supervision experiences for the participants as both supervisees and supervisors. Participants interviewed each other to practise the skills associated with building a student supervisory relationship and to articulate the skills they aspired to develop as a result of the training. Participants reported they wanted to develop skills in providing constructive feedback, supervising underperforming students, and discussing the possibility of a fail grade with a student. In addition, as the majority were currently supervising or intending to supervise staff, they expressed a desire to transfer skills learnt in the student supervision training to their supervision of staff. A variety of simulated activities provided participants with the opportunity to practise in these areas. After each exercise, participants were provided with structured feedback by their peers and the trainers on how to improve their approach. The use of power and authority and working with diversity were threaded throughout the day via discussion, information, and practice sessions.

This expanded version of Proctor’s model was presented in a way that optimised its utility for this cross disciplinary group. As the training was focussed on student supervision, changes were made to the commonly-used language, for example, the term ‘assessment’ was incorporated within the normative function and ‘wellbeing’ was employed to describe the restorative function. To facilitate clarity, the three supervisory functions were introduced separately, including the use of tools appropriate to each function. Consideration was then given to the inherent challenges and tensions involved in applying all three simultaneously in student supervision.

The restorative/supportive function was explained in terms of proactive self-care strategies. Participants explored issues such as managing professional boundaries, dealing with emotionally-charged situations, and responding to personal disclosures within supervision. The focus on the formative/educative function explored differences in learning styles and their relevance in the student supervision context. Specific attention was paid to difference and how this can offer both opportunities and challenges in the supervisory relationship. Discussions about the normative/accountability function explored benchmarks for student performance, reporting, and assessment requirements of educational institutions and professional standards and ethics.

The challenges associated with the simultaneous application of the three functions were explored though practice exercises using scenarios and through feedback from peers and trainers. These included examples of concerns about a student’s acquisition of the necessary professional skills, his/her risk of failure, or an experience of distress associated with clinical practice. These practice exercises addressed participants’ concerns around the provision of authentic and clear feedback, while illustrating how student performance relates to all three functions of supervision. The exercises and associated discussions facilitated development of the skills necessary to address each function and balance the inherent tensions between them.

### The evaluation framework

In total, 94 participants working in the Western Australian health sector attended the five one-day workshops in the Perth metropolitan area in 2013. All participants were approved and funded by the WADoH. Attendance varied from 11 to 26 participants, with a mean attendance of 18 people. Approval to conduct this research was provided by Curtin University’s Human Research Ethics Committee.

Participants were invited to complete pre- and post-evaluation forms anonymously (12 and 13 questions, respectively) on the day of training. Questions on the pre-evaluation form elicited quantitative and qualitative data which related to work and demographic profiles (qualifications, education, disciplinary background, employer-type, area of work, type of position held, years working in health, number of staff and students supervised), self-rating of knowledge, skills and values in student clinical supervision, and motivation to take up the training. Similarly, questions on the post-evaluation form elicited both quantitative and qualitative data and participants were invited to rate and comment upon various aspects of the training, the suitability and relevance of the supervision model, changes in their skills, knowledge, and values, their motivation, and the likelihood of accepting students in the future.

Ninety of the 94 participants completed the pre- and post-evaluation forms culminating in a response rate of 96%. A small number of respondents left some questions unanswered. All raw data were reviewed and entered by an independent statistician and then analysed using descriptive statistics. In order to determine if there were any differences of significance between characteristics, such as disciplines and length of time working in health, χ^2^ analyses were used.

## Results

### Profile of participants

Of the 90 participants who completed the pre- and post-evaluation forms, 94% were tertiary-educated. A variety of professional backgrounds were represented with occupational therapists most common (n = 26) followed by social workers (n = 17) and nurses (n = 13) (Table 3). Two-thirds of respondents were state government employees, 19% were private employees, 13% were from non-government organizations, and 2% were from local government; 27% of participants worked in acute care and the HWA priority areas of mental health (19%), community care (17%), and rehabilitation (16%) were also represented (Table 4).

**Table 3:** **Professional background of respondents**

Professional background		
	n	%
Occupational therapist	26	29
Social worker	17	19
Nurse	13	15
Physiotherapist	11	12
Speech pathologist	5	6
Other	5	6
Dietician	3	3
Podiatrist	3	3
Psychologist	3	3
Doctor	2	2
Pharmacist	1	1
Mental health (cert)	1	1

**Table 4:** **Employers and areas of work of respondents**

	n	%
**Employer**		
State government	59	66
Private	17	19
Non-government organization	12	13
Local government	2	2
Area of work*		
Acute health care	25	27
Mental health	18	19
Community health	16	17
Rehabilitation	15	16
Other	10	11
Disability	7	7
Palliative care	2	2
Primary health care	1	1

*Multiple responses allowed.

Thirty-nine respondents (43%) identified themselves as service-provider clinicians and were not currently supervising staff, 47% were combined service-provider clinicians and staff supervisors, and 6% identified with all three roles of service-provider clinician, staff supervisor, and manager. Length of service in the health sector varied with a third (32%) working for less than three years, and 40% working for more than 10 years.

In terms of supervisory roles in the workplace, 78% of respondents had supervised students in the last 12 months and 54% had supervised staff. Of the student supervisors, over a third (34%) had supervised five or more students. The number of years working in health had no bearing on whether respondents had provided clinical supervision to students in the last 12 months (*P* >0.05).

Given the inter-professional nature of the group, the data were segmented and analysed by professional groupings; these were occupational therapy (n = 26), social work (n = 17), nursing (n = 13), physiotherapy (n = 11), and all remaining allied health professionals (n = 23). The five groups were examined for any differences. Of note, a significantly greater proportion of occupational therapists, social workers, and physiotherapists were currently supervising no staff compared to nurses and other workers (χ^2^ = 20.176, *P* <0.001). A significantly higher proportion of social workers had worked in the industry for more than 6 years compared to the other four groups (χ^2^ = 15.461, *P* = 0.004).

### Skills, knowledge, and values

In spite of most respondents (80%) rating their level of supervisory skills pre-workshop as either ‘good’ (71%) or ‘very good’ , the majority (83%) reported that their skills had ‘definitely changed’ (41%) or ‘mostly changed’ (42%) post-training. Respondents indicated that their skill-enhancement included improvement in relationship skills, responsiveness to different learning styles, provision of feedback, placement planning, management of emotional and self-care issues, time management skills, and overall confidence. While most respondents (72%) rated their levels of knowledge pre-training as ‘good’ or better, 80% reported their knowledge had ‘definitely’ (40%) or ‘mostly changed’ (40%) as a result of the training. They reported their knowledge had improved in the following: clarification of the three supervision functions, awareness of student needs, clarification of professional boundaries, and the need for structure in student supervision. In relation to changes in values, 54% of the respondents indicated that their values had ‘definitely’ or ‘mostly’ been influenced by the training, particularly in terms of understanding the need to take a non-judgemental approach, having heightened empathy, appreciating difference, maintaining openness to positive and critical feedback, possessing an awareness of power differentials in supervision, and recognizing the importance of authentic dialogue. No statistically significant differences were found across the five professional groupings in regard to perceived levels of change in both skills and knowledge (*P* >0.05).

### The relevance and suitability of Proctor’s model

Eighty-eight percent of respondents reported that the extended Proctor’s model was both ‘definitely relevant and suitable’ (50%) or ‘mostly relevant and suitable’ (38%) (Table 5). A significantly greater proportion of social workers, compared to the other four professional groupings, reported that the model was definitely relevant and suitable (χ^2^ = 11.243, *P* = 0.004). This may reflect the model’s social work origins and the higher likelihood of theoretical or experiential exposure for this profession. Sixty-eight respondents offered positive comments, asserting that that the model was empowering and flexible, provided structure and a framework, was appropriate for students and staff, could be applied inter-professionally, and focussed on skills in balancing the three supervision functions. Levels of supervisory experience had no significant bearing on whether the supervision model was considered relevant (*P* >0.05).

**Table 5:** **Relevance and suitability of Proctor’s model by professional background**

Professional background	n	Definitely relevant %	Mostly relevant %	Unsure
Occupational therapist	26	40	32	28
Social worker	17	87	12	0
Nurse	13	46	46	7
Physiotherapist	11	30	60	10
Speech pathologist	5	60	40	0
Other	5	20	80	0
Dietician	3	0	100	0
Podiatrist	3	33	33	33
Psychologist	3	66	33	0
Doctor	2	50	50	0
Pharmacist	1	100	0	0
Mental health (cert)	1	100	0	0
Total	90	50	38	12

An experienced health worker with extensive staff and student supervisory experience reported that the supervision model created: “*a balance of all three areas, in particular, ensuring support and wellbeing of students is an essential part of being a good supervisor”.* A participant with no supervisory experience said: “*it gave an example of a supervision model that I can use. It reinforced my need to be self-aware of my actions/role. It gave me tools and skills”.*

### Motivation and future student supervision

The main motivations for choosing to participate in the training were the desire to increase knowledge, skills, and confidence, provide quality supervision, manage complex supervisory arrangements, provide constructive feedback, and meet requirements for promotion.

Respondents were asked whether they felt the training increased or decreased their likelihood of either starting or continuing student supervision and no statistically significant difference was found across the five professional groupings. However, 74% of all respondents indicated the training had ‘definitely increased’ (48%) or ‘mostly increased’ (26%) the likelihood (Table 6). Respondents explained that this was due to feeling empowered, confident, and enthusiastic; being more comfortable in the role of supervisor and having increased knowledge and skills. Six percent of respondents indicated the training had definitely not improved their likelihood of providing future student supervision and a further 20% indicated they were unsure or only minimally likely to take on future student supervision. These respondents commented: “*The number of students I supervise is determined by my company and the uni;* [it is] *out of my hands;* [there will be] *no real change; and* [we have] *minimal choice in whether we accept students or not*”.

**Table 6:** **Likelihood of starting or continuing student supervision by professional background**

Professional background	n	Definitely increased %	Mostly increased %	Unsure %	Minimally increased %	Definitely not increased %
Occupational therapist	26	29	33	19	19	0
Social worker	17	55	46	0	0	0
Nurse	13	64	9	18	9	0
Physiotherapist	11	67	22	0	11	0
Speech pathologist	5	50	25	0	25	0
Other	5	60	0	0	0	40
Dietician	3	0	0	0	0	100
Podiatrist	3	33	33	0	33	0
Psychologist	3	0	100	0	0	0
Doctor	2	100	0	0	0	0
Pharmacist	1	100	0	0	0	0
Mental health (cert)*	1	0	0	0	0	0
Total	90	48	26	9	11	6

*Did not complete.

## Discussion

The significance of this research is two-fold. First, it reports on the utility of an expanded version of Proctor’s model of supervision across different allied health professions and demonstrates respondents’ enthusiasm to develop skills and ensure their supervision is competent. Second, it considers the impact of HWA policy and funding on increasing the number and quality of student placements across Australian health professions and offers some qualifications.

The training program was rated highly by the vast majority of respondents. This suggests that the expanded version of Proctor’s model together with the experiential nature of the training suited health workers from a broad range of professional backgrounds, employers, workplace roles, length of service in the health sector, and levels of supervisory experience. Over 11 professions were represented, which distinguishes this project from the usual discipline-specific training. It is significant that no other example of the application of Proctor’s model in either staff or student clinical supervision training provided in an inter-professional context was found in the literature. Since there is widespread interest in staff and student learning afforded by multi-professional health settings, more research in this area is needed. However, whilst this training was rated highly, it is also important to note that evaluation is required to compare its efficacy to other forms of supervision training, particularly in light of HWA’s funding of a large number of clinical supervision training programs across Australia.

There was good uptake from the HWA priority areas of mental health, community care, and rehabilitation as well as one-third of respondents working in the emerging priority sectors of private or non-government organizations. With 43% of respondents having no prior experience of providing clinical supervision, the pool of clinical supervisors and placement opportunities across the health workforce has potentially grown. Given a third of respondents had been in the health workforce for three years or less, this also suggests that the training successfully targeted workers new to health. However, it is imperative that any actual increased numbers of both placements and competent supervisors afforded by training of this kind is assessed with follow-up studies. In addition, whether training such as this might have a longer term impact on retention and recruitment of staff also requires investigation.

A cautionary note needs to be sounded. Enhanced capacity and capability in student supervision and placement opportunities is only likely when all three of the HWA:CSSP foci of quality, clarity, and culture are attended to. As noted earlier, the goals of quality clinical supervision focus on accountability, ongoing professional development and promoting the wellbeing of the practitioner and it is important that the HWA drive to expand placements and numbers of qualified supervisors does not compromise on quality of clinical supervision in the workplace. In the cultural domain, supportive workplace environments and a critical mass of motivated supervisors are particularly important. While participants were keen volunteers for the clinical supervision training and the vast majority reported feeling motivated to supervise students in the future, there was also an uncertain or less motivated minority (26%), some of whom identified as conscripts to the role of supervisor. When compulsion exists, real cultural change in the health workforce is likely to be compromised and newly-harnessed motivation to provide quality student supervision experiences may dissipate.

Finally, it was not possible in this research to measure the medium and longer term impacts of this significant investment of government resources. We recommend that follow-up studies are conducted in the medium term to examine the effectiveness of such training on increasing numbers of clinical placements and improving the quality of clinical supervision and that, in the longer term, the impact of such training on recruitment and retention is also examined.

## Conclusions

It is established in the literature that quality clinical supervision has positive impacts on students and staff and that it can, in part, address the availability of student placements and competent supervisors. Trialling an innovative model of clinical supervision training for student supervisors across a range of health professions, which emphasises working with difference and power relations, is without precedent and the evaluation results suggest its suitability for inter-professional contexts. Future research investigating any differences across the professions will be important, as will longer term follow-up in terms of actual impact on placement numbers and availability of competent supervisors. Participants particularly valued the incorporation of the three functions of supervision simultaneously. They were also impressed by the flexibility and simplicity of the framework, its relevance and suitability for both students and staff, and the recognition of and work with power differentials and diversity. It will be important to test the expanded version of this model, which originates in social work, with other inter-professional cohorts for its impact on competencies across the professions.

In conclusion, these favourable first impressions and an expressed willingness by participants to adopt this clinical supervision model suggest that such training may hold the potential to increase the availability of quality clinical placements in a range of sites of health practice. Furthermore, these changes may be strengthened by the implementation of the cultural foci of the CSSP, where a systemic approach is taken to fostering a culture of quality clinical supervision within health service organizations. Longitudinal research is required in Australia to substantiate the extent to which the intention and motivation to supervise students garnered by training translates into sustainable increases in clinical placements and the supervision of students and staff across the health workforce. Research is also needed to ascertain if clinical supervision training such as this can elicit positive workforce effects such as enhanced recruitment and retention in the longer term.

## Authors’ information

Sue Gillieatt (BSc, DipEd, BSW, MA, DCA), Senior Lecturer, School of Occupational Therapy and Social Work, Faculty of Health Sciences, Curtin University. Robyn Martin (BSW, MSocSci, PhD), Lecturer, School of Occupational Therapy & Social Work, Faculty of Health Sciences, Curtin University. Trudi Marchant (BA, BSW, MHumanServ (Mgt&Policy)), Research Officer, School of Occupational Therapy & Social Work, Faculty of Health Sciences, Curtin University. Angela Fielding (BA, BSocWk, PhD), Director of Research Training and Associate Professor, School of Occupational Therapy & Social Work, Faculty of Health Sciences, Curtin University. Kate Duncanson (BSW), Director of Fieldwork, School of Occupational Therapy and Social Work, Faculty of Health Sciences, Curtin University.

## Abbreviations

CSSP: Clinical Supervision Support Program
Curtin: Curtin University
HWA: Health Workforce Australia
WADoH: Western Australian Department of Health.
